# Safety of COVID-19 vaccination in women undergoing IVF/ICSI treatment - Clinical study and systematic review

**DOI:** 10.3389/fimmu.2022.1054273

**Published:** 2023-01-11

**Authors:** Huijun Chen, Xiaoli Zhang, Ge Lin, Fei Gong, Berthold Hocher

**Affiliations:** ^1^ Fifth Department of Medicine (Nephrology/Endocrinology/Rheumatology/Pneumology), University Medical Centre Mannheim, University of Heidelberg, Mannheim, Germany; ^2^ Clinical Research Center for Reproduction and Genetics in Hunan Province, Reproductive and Genetic Hospital of CITIC-Xiangya, Changsha, Hunan, China; ^3^ Institute of Pharmacy, Freie Universität Berlin, Berlin, Germany; ^4^ Institute of Reproductive and Stem Cell Engineering, NHC Key Laboratory of Human Stem Cell and Reproductive Engineering, School of Basic Medical Science, Central South University, Changsha, Hunan, China; ^5^ Key Laboratory of Stem Cells and Reproductive Engineering, Ministry of Health, Changsha, China; ^6^ Institute of Medical Diagnostics, IMD, Berlin, Germany

**Keywords:** COVID-19, vaccine, IVF/ICSI, fertilization rate, pregnancy outcomes

## Abstract

**Background:**

It was suggested that vaccination in general might affect reproductive health. Safety of COVID-19 vaccination in women undergoing assisted reproductive techniques (ART) treatment is not well established.

**Methods:**

We performed a retrospective study including 536 women undergoing fresh embryo transfer after IVF/ICSI treatment in a huge IVF center in southern China to investigate the effect of COVID-19 vaccination on oocyte maturation, fertilization rate, blastulation rate, implantation rate, clinical pregnancy rate and miscarriage rate. In addition, we performed a systematic review of existing studies on the safety of COVID-19 vaccination in women undergoing ART treatment.

**Results:**

In our study, 268 women received inactivated or recombinant COVID-19 vaccination and 268 controls were enrolled based on propensity score matching. We observed a decreased fertilization rate and signs for impaired oocyte maturation in vaccinated women. Besides our study, there were 15 studies analyzing the safety of COVID-19 vaccination in women undergoing ART treatment. For the mRNA vaccines, no adverse signals were reported concerning oocyte maturation, fertilization rate, blastulation rate, implantation rate, clinical pregnancy rate and miscarriage rate. In women being vaccinated with an inactivated vaccine, implantation rate, clinical pregnancy rate and miscarriage rate were not affected, whereas oocyte maturation and fertilization rate were impaired.

**Conclusions:**

Vaccination against COVID-19 in women undergoing ART treatment seems to be safe especially for women getting mRNA vaccines. The effects on oocyte maturation and fertilization rate of inactivated and recombinant COVID-19 vaccinations might be a safety signal and need further investigation and independent confirmation.

## Introduction

The appearance and rapid spread of the novel coronavirus disease 2019 (COVID-19) in 2020 have caused loss of life and poorer health outcomes also in pregnant women and their newborns. The SARS-CoV-2 virus belongs to the β-corona virus family as the other human pathogenic corona viruses. In patients with a more severe upper respiratory tract infection, the SARS CoV-2 infection often causes a mild and/or severe acute respiratory syndrome (SARS) with subsequent release of cytokines/mediators such as: interleukin (IL)-1β, IL-2, IL-4, IL-5, IL-6, IL-7, IL-8 (CXCL8), IL-10, IP10, IL-12, IL-13, IL-17, IL-33, IL-25, IL-37, IL-38, GCSF, GM-CSF, HGF, IP-10, MCP-1, MIP-1α, IFN-γ, IFN-α, TRAIL, MCSF, and TNF-α (1). There is clear evidence suggesting that SARS-CoV-2 may infect the ovary, uterus and vagina because these organs do express angiotensin converting enzyme 2 (ACE2), the key host factor for SARS-CoV2 infection (2, 3). SARS-CoV-2 infection was shown to adversely affect female reproductive functions (luteal angiogenesis and degeneration, regular changes in endometrial tissue, and embryo development) and increased the risk of infertility and menstrual cycle disorder (4–6). Infection with SARS-CoV-2 results in a down-regulation of ACE-2 expression, subsequentially leading to reduced conversion of angiotensin (ANG)-II to Ang 1–7 and excessive accumulation of ANG-II which causes for example reactive oxygen species (ROS) production. This alters ovarian steroidogenesis, follicular development, oocyte maturation, ovulation, and atresia (7). Moreover, SARS-CoV-2 infection, often causing an excessive production of proinflammatory cytokines, including IL6, IL1β, TNFα, and MCP-1/CCL2, impairs reproductive physiology, such as ovulation and endometrial receptivity (7). It was suggested that Ang II mediated excessive ROS production might reduce female fertility (8). Moreover, pregnant women with symptomatic COVID-19 were more likely to experience ICU admission, intubation, and death (9). SARS CoV-2 infection seems to be associated with oligohydramnios, fetal growth restriction, preterm birth, and stillbirth (10–14) and potential long-term adverse health outcomes (15). Since there is no precise cure for the disease in pregnant women approved so far, mass vaccination is a key method by which countries are aiming to control the pandemic for this special population (16).

Due to safety reasons, however, pregnant and breastfeeding women were not included in the initial Phase III clinical trials of the COVID-19 vaccine developmental programs, resulting in limited data on the efficacy and more important safety of the COVID-19 vaccine in pregnant and breastfeeding women (17). Since the end of 2020, key national and international health institutions like the WHO as well as academic associations guided the COVID-19 vaccine, including women who are pregnant, breastfeeding, or planning pregnancy (naturally or with the help of assisted reproductive technology) (18). Antibody titers after vaccination are similar in pregnant and non-pregnant women (19). A large prospective cohort study including 7809 pregnant women found that COVID-19 vaccines were well tolerated in pregnant and lactating women (20). A systematic review including 26 studies analyzing perinatal outcomes after COVID-19 vaccination in pregnancy showed that the overall rates of adverse perinatal outcomes were not increased after maternal vaccination (21). However, there is still an ongoing controversial debate on the safety and efficacy of COVID-19 vaccination in this population (22).

Bentov et al. assessed the follicular steroidogenesis and oocyte quality in women undergoing mRNA vaccination and did not see any difference when compared with unvaccinated women (23). For *in-vitro* fertilization (IVF) treatment, a study reported that COVID-19 infection could lead to a significantly lower proportion of top-quality embryos while no difference in the number of oocytes and mature oocytes retrieved, and fertilization rate was seen. However, the sample size in this study was small and for sure beyond the power to seriously address safety concerns (24).

For the prevention of COVID-19, several vaccine types have been developed and approved so far (inactivated virus vaccine, mRNA vaccine, live attenuated vaccine, recombinant protein subunit vaccine, and vector vaccine) (25). Whether a different type of vaccine affects reproduction outcomes is unknown. Since the IVF procedure is a unique controlled process of human reproduction, the effects of vaccination on the different steps of human reproduction (oocyte maturation, fertilization, embryo development, implantation, biochemical pregnancy, clinical pregnancy, early and late abortion, and finally life birth rate) can be distinguished. We thus compared in a retrospective study the effects of two main types of vaccination on pregnancy outcomes. We compared inactivated and recombinant COVID-19 vaccines:

A) Inactivated vaccine, which used Vero cells to culture and grow COVID-19 virus and β-propionolactone to inactivate the virus while retaining antigenic components that induce immune responses (26).
B) Recombinant protein vaccine, which is based on recombined coronavirus S protein receptor-binding region (RBD) gene expression in Chinese hamster ovary (CHO) cells. The CHO cells make RBD protein dimers that are purified and concentrated for use as a vaccine. Aluminum hydroxide adjuvant is added to improve immunogenicity (26).

In addition, we performed a systematic review of all available information’s on the safety of the different COVID-19 vaccines during pregnancy with a focus on reproductive medicine safety endpoints.

## Materials and methods

### Study design and setting

This is a retrospective analysis of women who received ART treatment from June 2021 to September 2021 in the Reproductive and Genetic Hospital of CITIC-Xiangya. 536 women were enrolled in the analysis by propensity score matching (PSM): women received COVID-19 vaccination (n = 268) and control group (n = 268). (for more details see: **Supplementary Figure S1**). The vaccines used are described in detail in **Supplementary Table S1**. Based on the vaccine type we build two vaccination groups and a control group without any COVID-19 vaccination.

The study was approved by the Ethics Committee of the Reproductive and Genetic Hospital of CITIC-Xiangya (approval number: LL-SC-2022).

### Participants

The participant’s inclusion and exclusion criteria were as follows:

Inclusion criteria: (1) age: 20-45 years, (2) women received ovarian stimulation and oocyte retrieval due to any type of infertility. Exclusive criteria: (1) women receiving oocyte donation, (2) women undergoing frozen embryo transfer cycles, and (3) prior SARS-CoV-2 infection. (4) male partners received any COVID-19 vaccination or had SARS-CoV2 infection.

### Outcome measurement

The primary study endpoints were: normal fertilization rate and clinical pregnancy rate, For the primary study endpoints multivariant linear regression analysis considering confounding factors were done.

All other study outcomes such as laboratory values, oocyte and embryo quality data were analyzed as described recently (27). Normal fertilization rate was defined as

Normal fertilization rate was defined as the presence of two pronuclei (2PN) 16-18 hours after fertilization, basically one from sperm and the other from oocyte. Clinical pregnancy was defined as the existence of gestational sac(s) with fetal heart activity by ultrasound at week 4 after ET. Implantation rate was defined as the total number of embryos transferred divided by the number of sacs. Miscarriage was defined as intrauterine pregnancy loss after confirmation of gestational sacs.

### Data analysis

We applied Statistical Package for Social Sciences for Windows, version 25.0 (SPSS Inc, Chicago, IL, USA) to perform data analyses. Homogeneity of variance and normality of data was estimated using the Levene and Kolmogorov-Smirnov tests, respectively. Values were expressed as means ± standard deviation or frequency (%). A comparison of quantitative variables between groups was performed using the Mann-Whitney U test or T-test. Qualitative variables were compared by the Chi-square test or Fisher’s exact test. Multivariate logistic regression analysis (enter model) and multiple linear regression analysis (kick-out model) were performed to figure out the impact factor on pregnancy and fertilization rate. Baseline clinical factors related to fertility were considered confounding factors. Statistically significant data was indicated with p < 0.05.

### Systematic review

**Figure 1:**
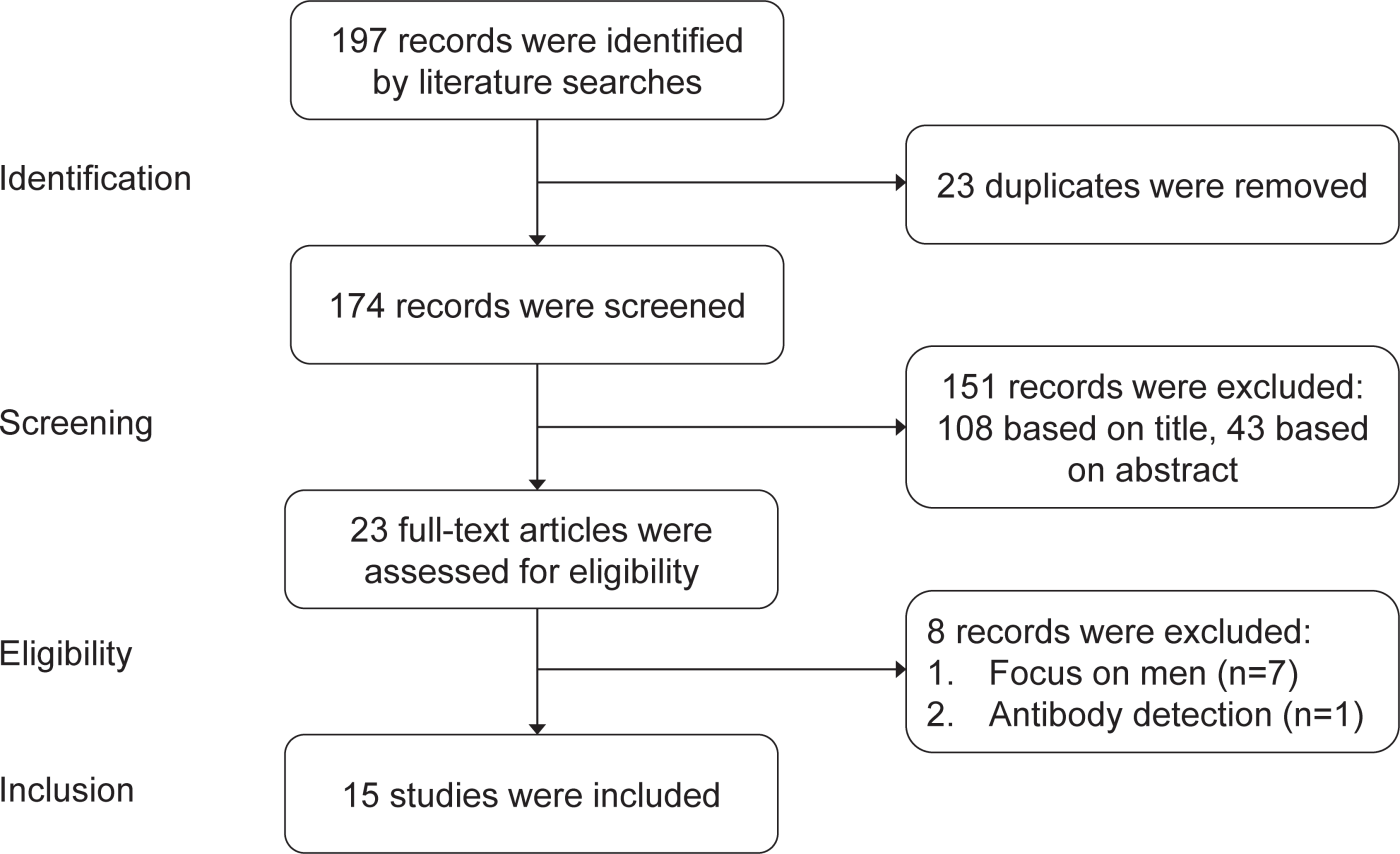
Flow chart of screening studys into systematic review.

The systematic review of the safety of COVID-19 vaccines in embryo transfer included studies published from January 1^st^, 2021 to December 1^st^, 2022. We focused on studies published in English. We used the following sources: PubMed, Google scholar, Embase and Scopus. The search terms were ‘COVID-19 vaccine’ OR ‘COVID-19 mRNA vaccine’ OR ‘COVID-19 inactivated vaccine’ AND ‘embryo transfer’ OR ‘IVF outcomes’ OR ‘ICSI outcomes’ OR ‘pregnancy outcomes’. Our literature searches yielded 197 studies, from which 23 duplicates were removed. After a review of the titles and abstracts, 23 studies were identified as potentially eligible for inclusion. 7 studies focused on men instead of women and 1 study focused on the antibody after COVID-19 vaccination. Finally, 15 studies were included in the systematic review. See details in **Figure 1**.

## Results

### Clinical findings

We matched the vaccinated women and controls by PSM (propensity score matching) (**Supplementary Figure S1**). Next, we analyzed the different types of vaccines separately. In the present study, most of our participants (n=223) received an inactivated vaccine, 44 women received a recombinant vaccine and only 1 woman received an adenovirus vector vaccine, which was excluded from further analysis. Information’s of the used vaccine is shown in **Supplementary Table S1**. For women who received the inactivated vaccine, baseline anti-Müllerian hormone (AMH) concentrations were lower as compared to the controls and those who received the recombinant vaccine (**Table 1**). No other differences were observed in other baseline characteristics among groups, see **Table 1**.

**Table 1:** The demographic and clinical characteristics of participants.

	Control (n=268)	Inactivated vaccine (n=223)	Recombinant vaccine (n=44)	P^a^	P^b^	P^c^
Age (y)	32.81 ± 4.92	33.32 ± 5.14	33.00 ± 5.16	0.26	0.81	0.70
Infertility type (%)						
Primary	29.85 (80)	31.84 (71)	45.45 (20)	0.08	0.06	0.22
Secondary	61.57 (165)	64.57 (144)	52.27 (23)			
Others	8.58 (23)	3.59 (8)	2.27 (1)			
Infertility reason *						
Fallopian tube malfunction	214	170	33			
Ovulation dysfunction	5	2	1			
Endometriosis	16	16	5			
Intrauterine adhesion	73	84	10			
Chronic endometritis	30	36	10			
Genetic abnormality	30	18	2			
Male reason	11	15	3			
BMI (kg/m2)	21.98 ± 2.56	21.94 ± 2.51	22.45 ± 2.65	0.86	0.27	0.25
Waist circumference (cm)	73.78 ± 7.81	72.86 ± 7.12	74.80 ± 8.09	0.10	0.52	0.11
Hip circumference (cm)	91.33 ± 6.59	90.96 ± 5.88	92.59 ± 7.04	0.51	0.25	0.11
Waist-to-hip ratio	0.81 ± 0.07	0.80 ± 0.05	0.81 ± 0.06	0.09	0.84	0.42
Infertility interval (y)	3.69 ± 3.35	3.55 ± 3.38	3.48 ± 2.35	0.65	0.69	0.90
AMH (ng/ml)	4.34 ± 3.40	3.62 ± 3.59	5.15 ± 5.56	0.02	0.36	0.02
AFC	22.50 ± 16.43	19.93 ± 16.85	28.45 ± 28.53	0.09	0.18	0.06
FSH (mIU/ml)	6.78 ± 3.08	7.09 ± 3.01	7.40 ± 4.42	0.27	0.25	0.56
LH (mIU/ml)	4.73 ± 3.08	4.38 ± 2.84	4.88 ± 3.47	0.19	0.77	0.31
PRL	18.63 ± 26.33	16.64 ± 9.04	21.48 ± 25.78	0.28	0.51	0.23
E2 (pg/ml)	49.18 ± 63.82	54.39 ± 101.68	58.02 ± 67.03	0.49	0.40	0.77
P (ng/ml)	1.29 ± 3.36	1.24 ± 3.38	1.31 ± 2.82	0.87	0.97	0.88
T (ng/ml)	2.42 ± 9.69	2.05 ± 7.41	0.90 ± 3.26	0.64	0.31	0.10
Sperm quality						
Before processing						
Volume (ml)	1.14 ± 0.40	1.17 ± 0.53	1.13 ± 0.51	0.49	0.86	0.63
Density (x10^6/ml)	46.80 ± 24.92	47.26 ± 23.88	47.02 ± 23.32	0.84	0.96	0.95
Motility (%)						
Grade A	7.78 ± 3.46	7.71 ± 3.38	7.43 ± 3.19	0.82	0.52	0.61
Grade B	21.65 ± 9.47	21.40 ± 9.06	22.41 ± 8.27	0.77	0.62	0.49
Grade C	14.03 ± 9.74	13.87 ± 7.18	15.16 ± 7.58	0.84	0.46	0.28
Grade D	46.21 ± 17.96	48.18 ± 38.56	61.83 ± 93.70	0.46	0.28	0.11
After processing						
Volume (ml)	0.18 ± 0.09	0.17 ± 0.07	0.16 ± 0.06	0.02	0.07	0.52
Density (x10^6/ml)	16.03 ± 7.88	16.35 ± 7.38	16.77 ± 6.98	0.64	0.56	0.95
Motility (%)						
Grade A	32.99 ± 12.34	32.02 ± 12.31	34.06 ± 10.76	0.39	0.59	0.31
Grade B	50.09 ± 18.47	50.47 ± 19.01	52.30 ± 15.68	0.82	0.45	0.55
Grade C	2.53 ± 4.06	2.24 ± 2.06	2.67 ± 2.04	0.33	0.83	0.21
Grade D	3.74 ± 2.82	3.97 ± 3.48	4.15 ± 3.21	0.46	0.38	0.75

All hormones were tested during the initial visits before initiating stimulation therapy.

*Women can have more than one infertility reason.

Sperm quality was accessed according to WHO criteria (28). Grade A, rapidly progressive motility; Grade B, slowly progressive motility; Grade C, sluggish motility; Grade D, immotile sperm.

a, inactivated vaccine vs. control; b, recombinant vaccine vs. control; c, recombinant vaccine vs. inactivated vaccine.

BMI, body mass index; AMH, anti-Müllerian hormone; AFC, antral follicle count; FSH, follicle-stimulating hormone; LH, luteinizing hormone; PRL, prolactin; E_2_, estradiol; P, progesterone; T, testosterone.

The primary study endpoints of our single center study were normal fertilization rate and clinical pregnancy rate.

**Figure 2:**
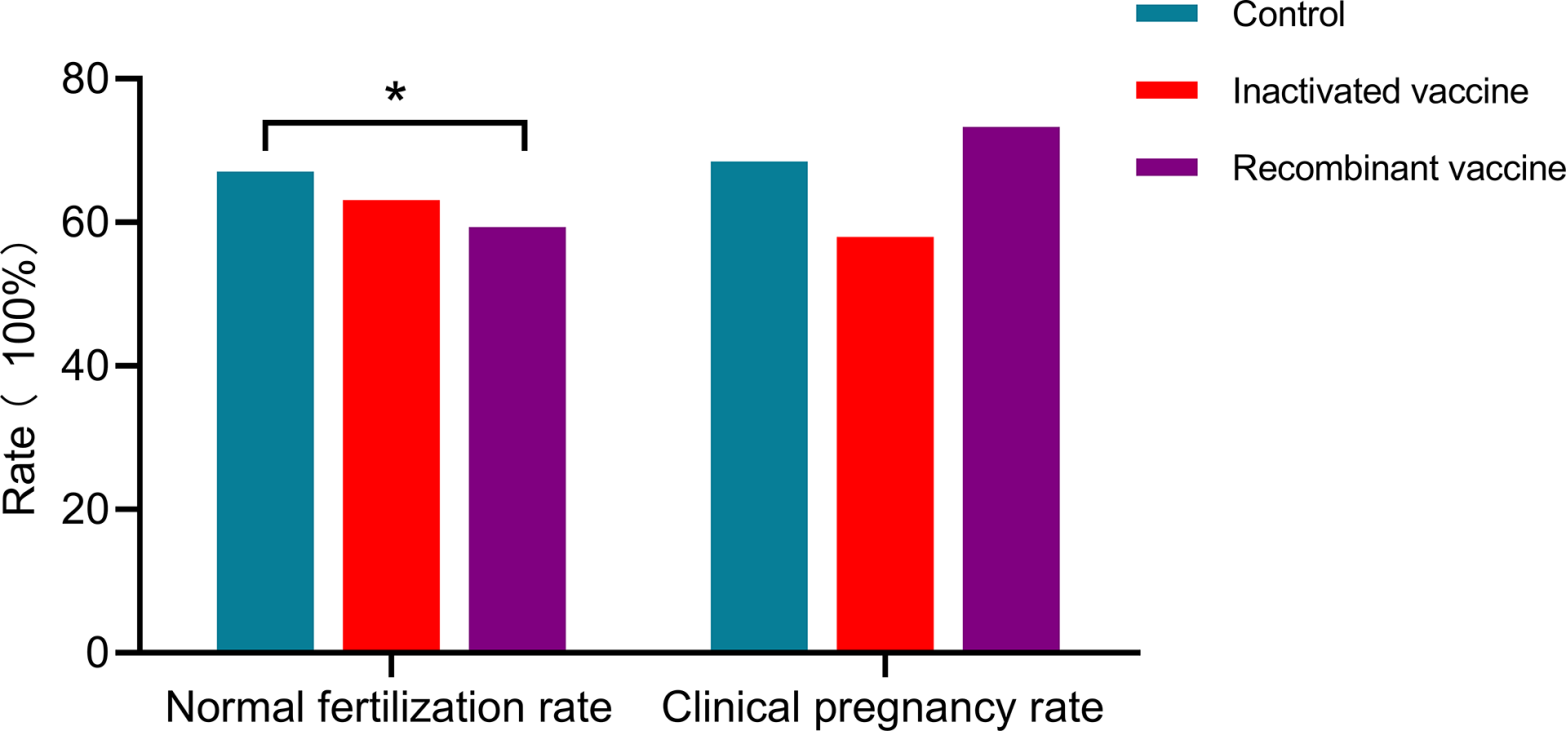
Preganncy outcomes in different vaccine type. * P<0·05.

In women received inactivated vaccine, there was no significant difference in both normal fertilization rate and clinical pregnancy rate compared to controls (**Tables 2**, **3**; **Figure 2**). Concerning the secondary outcomes, only the number of MI phase oocytes (immature oocytes) was higher than controls (**Table 2**). Since there is no consensus on the time interval after vaccination to receive ART treatment, we regrouped the participants according to a time cut-off determined by Receiver Operating Characteristic (ROC) analysis. The time cut-off value was six weeks. Hence, we grouped our participants into two groups: <six weeks and ≥ six weeks. Analysis of time-dependency (<six weeks versus ≥ six weeks) of potential effects of inactivated vaccine revealed no difference with regard to oocyte-, zygote- and embryo-quality and pregnancy outcome (**Supplementary Table S2**, **S4**). Multivariant linear regression analysis (normal fertilization rate) and logistic regression analysis (clinical pregnancy rate) considering confounding factors were performed. The results showed that inactivated vaccination had a negative impact on the normal fertilization rate (**Table 4**) but not on the clinical pregnancy rate (**Table 5**). There was no time dependency of the adverse effects of inactivated vaccination on normal fertilization rate and clinical pregnancy rate (**Tables 4**, **5**).

**Table 2:** Laboratory outcomes in women received different type of vaccines and controls.

	Control (n=268)	Inactivated vaccine (n=223)	Recombinant vaccine (n=44)	P^a^	P^b^	P^c^
No. of oocytes retrieved	10.40 ± 5.98	10.15 ± 6.76	12.48 ± 8.33	0.67	0.05	0.05
No. of degenerated oocytes	0.07 ± 0.29	0.07 ± 0.27	0.09 ± 0.29	0.89	0.67	0.60
No. of GV phase oocytes	0.66 ± 1.23	0.62 ± 1.17	0.80 ± 2.25	0.70	0.56	0.45
No. of MI phase oocytes	0.39 ± 0.85	0.61 ± 1.47	0.77 ± 1.20	0.05	0.04	0.48
No. of MII phase oocytes	9.28 ± 5.38	8.86 ± 6.13	10.82 ± 7.68	0.42	0.21	0.07
Fertilization methods						
IVF	56.88 (153)	61.43 (137)	70.45 (31)	0.22	0.22	0.36
ICSI	38.06 (102)	31.39 (70)	27.27 (12)			
IVF+ICSI	4.85 (13)	7.17 (16)	2.27 (1)			
Normal fertilization rate (%)	67.13 (1871/2787)	63.07 (1428/2264)	59.38 (326/549)	0.15	0.01	0.11
No. of 1PN zygotes	0.35 ± 0.64	0.38 ± 0.59	0.50 ± 0.82	0.69	0.04	0.24
No. of 2PN zygotes	8.27 ± 4.95	7.74 ± 5.42	9.25 ± 7.32	0.25	0.26	0.11
No. of ≥3PN zygotes	0.80 ± 1.30	0.60 ± 0.99	1.16 ± 1.46	0.06	0.08	0.02
D3 embryo quality*						
Fair	4.64 ± 3.01	4.65 ± 3.86	4.84 ± 4.25	0.97	0.70	0.77
Good	3.47 ± 3.27	3.30 ± 3.34	3.73 ± 3.99	0.58	0.64	0.46
8C-I	1.15 ± 1.78	0.95 ± 1.45	1.50 ± 2.05	0.18	0.23	0.09
D3 good quality embryo rate (%)	42.78 (930/2174)	41.52 (737/1775)	43.50 (164/377)	0.43	0.79	0.48
Blastocyst formation rate (%)						
D5	36.15 (557/1541)	41.52 (479/1261)	37.46 (106/283)	0.31	0.67	0.87
D6	40.74 (486/1193)	39.57 (406/1026)	33.79 (74/219)	0.58	0.05	0.26
D7	25.76 (59/229)	29.15 (65/223)	27.78 (5/18)	0.42	0.85	0.90
Good quality blastocyst rate (%) **						
D5	14.36 (80/557)	16.49 (79/479)	18.87 (20/106)	0.34	0.24	0.56
D6	31.07 (151/486)	31.28 (127/406)	21.62 (16/74)	0.95	0.10	0.10
D7	38.98 (23/59)	29.23 (19/65)	60.00 (3/5)	0.25	0.66	0.35

GV, germinal vesicle; GV phase oocytes did not resume meiosis (immature oocytes); MI, metaphase I, oocytes in the middle of the first meiosis (immature oocytes); MII, metaphase II, oocytes in the middle of the second meiosis (mature oocytes); IVF, in vitro fertilization; ICSI, intracytoplasmic sperm injection; PN, pronucleus (only 2PN means normal fertilization); D5, day 5 after fertilization; D6, day 6 after fertilization; D7, day 7 after fertilization.

*D3 embryo quality, day 3 after fertilization. Fair quality embryo, embryo grade <7C-II; good quality embryo, embryo grade ≥7C-II.

**Good quality blastocyst, ≥4BB

a, inactivated vaccine vs. control; b, recombinant vaccine vs. control; c, recombinant vaccine vs. inactivated vaccine.

**Table 3:** Pregnancy outcomes in women received embryo transfer.

	Control (n=127)	Inactivated vaccine (n=77)	Recombinant vaccine (n=15)	P^a^	P^b^	P^c^
EM thickness before ET (mm)	12.58 ± 1.82	12.25 ± 1.89	12.46 ± 2.05	0.21	0.80	0.31
Mean number of embryos transferred	1.69 ± 0.47	1.69 ± 0.47	1.87 ± 0.35	0.96	0.14	0.10
Day3 embryo	1.95 ± 0.22	1.88 ± 0.33	1.92 ± 0.29	0.15	0.63	0.73
Blastocyst	1.21 ± 0.41	1.22 ± 0.42	1.25 ± 0.50	0.89	0.84	0.90
Good-quality embryo transferred rate (%)	77.57 (166/214)	80.00 (104/130)	75.00 (21/28)	0.59	0.76	0.55
Clinical pregnancy rate (%)	68.50 (87/127)	57.94 (46/77)	73.33 (11/15)	0.20	0.70	0.48
Implantation rate (%)	56.07 (120/214)	45.38 (59/130)	50.00 (14/28)	0.05	0.54	0.65
Miscarriage rate (%)	4.72 (6/127)	2.60 (2/77)	13.33 (2/15)	0.67	0.43	0.24

EM, endometrium; ET, embryo transfer.

Some women cancelled embryo transfer, the reasons were listed in **Supplementary Figure S1**

a, inactivated vaccine vs. control; b, recombinant vaccine vs. control; c, recombinant vaccine vs. inactivated vaccine.

**Table 4:** Multi liner regression analysis on normal fertilization rate (kick-out model).

A. All women received vaccination (either inactivated or recombinant vaccine)								
	Unadjusted		Adjusted	VIF	P value	95% CI		
	OR	Standard error	OR			Lower bound		Upper bound
Constant	0.569	0.030			<0.01	0.511		0.627
Vaccination(yes/no)	-0.062	0.021	-0.122	1.000	<0.01	-0.104		-0.020
Grade B sperm before processing	0.004	0.001	0.157	1.000	<0.01	0.002		0.007
B. Women received inactivated vaccine								
	Unadjusted		Adjusted	VIF	P value	95% CI		
	OR	Standard error	OR			Lower bound		Upper bound
Constant	0.593	0.026			<0.01	0.541		0.644
Vaccination(yes/no)	-0.052	0.022	-0.104	1.000	0.02	-0.096		-0.008
Sperm density before processing	0.001	0.000	0.145	1.000	<0.01	0.001		0.002
C. Women received recombinant vaccine								
	Unadjusted		Adjusted	VIF	P value	95% CI		
	OR	Standard error	OR			Lower bound	Upper bound	
Constant	0.638	0.041			<0.01	0.557	0.718	
Vaccination(yes/no)	-0.061	0.020	-0.173	1.001	<0.01	-0.100	-0.023	
Grade B sperm before processing	0.006	0.002	0.242	2.264	<0.01	0.002	0.011	
Grade A sperm after processing	-0.003	0.002	-0.170	2.264	0.04	-0.007	0.000	

Age, waist-to-hip-ratio, BMI, FSH, LH, E2, P, T, AMH, AFC, sperm quality before and after processing, vaccine type and time interval after vaccination to receive ART treatment were included as confounding factors. Only significant parameters of the final model are shown.

BMI, body mass index; FSH, follicle-stimulating hormone; LH, luteinizing hormone; E2, estradiol; P, progesterone; T, testosterone; AMH, anti-Müllerian hormone; AFC, antral follicle count; EM, endometrium.

**Table 5:** Multivariate regression analysis on clinical pregnancy rate (only women received embryo transfer).

A. All women received vaccination.					
	B	P value	OR	95% CI of OR	
				Lower bound	Upper bound
Age	-0.206	<0.01	0.814	0.744	0.891
EM	0.250	0.01	1.284	1.061	1.553
B. Women received inactivated vaccine					
	B	P value	OR	95% CI of OR	
				Lower bound	Upper bound
Age	-0.205	<0.01	0.815	0.743	0.893
EM	0.260	0.01	1.297	1.063	1.583
C. Women received recombinant vaccine					
	B	P value	OR	95% CI of OR	
				Lower bound	Upper bound
Age	-0.195	<0.01	0.823	0.734	0.923
Constant	6.414	0.09	610.275		

Age, waist-to-hip-ratio, BMI, FSH, LH, E2, P, T, AMH, AFC, sperm quality before and after processing, vaccine type and time interval after vaccination to receive ART treatment were included as confounding factors. Only significant parameters of the final model are shown.

BMI, body mass index; FSH, follicle-stimulating hormone; LH, luteinizing hormone; E_2_, estradiol; P, progesterone; T, testosterone; AMH, anti-Müllerian hormone; AFC, antral follicle count; EM, endometrium.

In women receiving recombinant vaccine, normal fertilization rate was lower as compared to controls (**Table 2**; **Figure 2**). Concerning the secondary outcomes, Women with recombinant vaccination received more retrieved oocytes, MI phase oocytes (immature oocytes) and one pronucleus (1PN) than controls (**Table 2**). Analysis of time-dependency (<six weeks versus ≥ six weeks) of potential effects of recombinant vaccine showed that the number of 1PN zygotes was significantly higher while the day five (D5) blastocyst formation rate and the day six (D6) good quality blastocyst rate were lower in women who received ART treatment less than six weeks after recombinant vaccination (**Supplementary Table S3**). Univariant not adjusted analysis showed that clinical pregnancy rate was higher in women received ART treatment more than six weeks after recombinant vaccination (**Supplementary Table S5**). Multivariant regression analysis showed that recombinant vaccination had a negative impact on the normal fertilization rate (**Table 4**) but had no impact on clinical pregnancy rate (**Table 5**). Time interval after recombinant vaccination to receive ART treatment had no effects on normal fertilization rate and clinical pregnancy rate (**Tables 4**, **5**).

Taken together, it appears that the inactivated vaccine might have adverse effects on oocyte maturation. This, however, does not substantially affect the primary study endpoints of our single center study: normal fertilization rate and clinical pregnancy rate

### Systematic review

We compared existing studies on the safety of COVID-19 vaccines in embryo transfer published from January 1^st^, 2021 to December 1^st^, 2022. According to the searching and screening criteria described in the method section, 15 studies were taken into account finally.

In these 15 studies, 2 studies from America reported the safety of mRNA vaccination and one of which involved adenovirus vector vaccines (29, 30), 1 study from Spain (31) and 7 studies from Israel (24, 32–37) mainly reported the safety of mRNA vaccination and 5 studies from China (38–42) reported the safety of inactivated vaccination and one of which also involved adenovirus vector vaccines and recombinant subunit vaccines.

#### Oocyte maturation

Nine studies analyzed oocyte maturation and there was no difference in oocyte maturation in all these studies (24, 30, 33–37, 40, 42). However, there are more MI oocytes in vaccinated women, especially in those who received the recombinant vaccine in our study.

#### Fertilization rate

Twelve studies analyzed the fertilization rate. One study (29) reported a higher fertilization rate in vaccinated women while other studies found no difference (24, 30, 32–36, 38–40, 42). In our present study, a lower fertilization rate was observed in vaccinated women, especially in those who received recombinant vaccine

#### Blastulation rate

Nine studies reported blastulation rate, no difference was observed in all the studies (29, 30, 33, 36, 38–42) and also in our study.

#### Implantation rate

Five studies reported implantation rate, no difference was observed in all the studies (31, 32, 39, 40, 42) and also in our study.

#### Clinical pregnancy rate

Twelve studies analyzed clinical pregnancy rate, no difference was observed in all the studies (30–34, 36–42) and also in our study.

#### Ongoing pregnancy rate

Six studies analyzed ongoing pregnancy rate. One study showed that ongoing pregnancy rate was significantly lower in women who received the first inactivated COVID-19 vaccine dose 60 days or less before fertilization treatment. However, no reduced risk for ongoing pregnancy in the 91 days or more subgroup was observed (42). No difference was observed in the other studies (29, 30, 32, 39, 41) and also in our study.

#### Miscarriage rate

Four studies analyzed miscarriage rate, no difference was observed in all the studies (29, 30, 39, 41) and also in our study.

In summary, almost all of these studies stated that vaccination had no detrimental effect on cycle characteristics or other laboratory and pregnancy outcomes in women undergoing ART treatment except that one study observed that vaccinated patients had higher fertilization rates than unvaccinated patients. However, in the current study, more MI oocytes and lower fertilization rate were observed in vaccinated women, especially in those who received recombinant vaccine. See more details in **Table 6**.

**Table 6:** Systematic review of COVID-19 vaccination studies in women undergoing IVF/ICSI treatment.

Study	Design	Region	Sample size	Vaccination type	Time interval from vaccination to ART	Cycles	Main findings	Reference
Emily Jacobs, et al. (2022)	Retrospective cohort study	America	142 vaccinated women and 138 controls	mRNA vaccine and adenovirus vector vaccines	93 ± 65 (mean ± SD) days	Fresh embryo transfer	Vaccinated patients had higher fertilization rates than unvaccinated patients. No significant differences in ovarian reserve, ovarian response, number of oocytes and retrieved useable embryos, ongoing clinical pregnancy rate and miscarriage rate.	(29)
Devora Aharon, et al. (2022)	Retrospective cohort study	America	222 vaccinated women and 983 controls	mRNA vaccine	N/A	Fresh cycle and frozen embryo transfer	No significant association between vaccination and clinical pregnancy or any of the secondary outcomes: ongoing pregnancy, biochemical pregnancy loss and clinical pregnancy loss in women undergoing IVF treatment.	(30)
Pedro Brandão, et al. (2022)	Retrospective cohort study	Spain	890 vaccinated women and 3272 controls	mRNA vaccine	Q1 = less than 1.8 months, Q2 = 1.8 to 3.1 months, Q3 = 3.2 to 4.5 months and Q4 = 4.5 months or more according to quartiles	Fresh and frozen embryo transfer	Vaccination had no effect on clinical pregnancy rate and sustained implantation rate, regardless of the number of doses and time interval from vaccination to embryo transfer.	(31)
Adva Aizer, et al. (2022)	Retrospective cohort study	Israel	141 patients (post-infected and vaccinated) and 287 controls	mRNA vaccine	N/A	Frozen embryo transfer	Vaccination did not affect patients’ performance or implantation in their subsequent frozen-thawed embryo transfer cycle.	(32)
Sarit Avraham, et al. (2022)	Retrospective cohort study	Israel	200 vaccinated women and 200 controls	mRNA vaccine	14–68 days	Fresh and frozen embryo transfer	No difference was noted in the fertilization rate, clinical pregnancy rates and transferred embryos’ quality between vaccinated and unvaccinated patients.	(33)
Raoul Orvieto, et al. (2021)	Observational study	Israel	36 couples	mRNA vaccine	7-85 days	N/A	Vaccination did not affect patients’ performance or ovarian reserve in their subsequent IVF cycle.	(24)
Rasha Odeh-Natour, et al. (2021)	Prospective, observational cohort study	Israel	37 vaccinated women and 22 controls	mRNA vaccine	2 weeks to 2 months	Fresh embryo transfer	Vaccination did not affect treatment outcomes or pregnancy outcomes in women undergoing IVF/ICSI cycles.	(34)
Gilad Karavani, et al. (2022)	Retrospective cohort study	Israel	83 vaccinated women, 55 intra-pandemic unvaccinated women and 133 pre-pandemic women	mRNA vaccine	1 to 13 months	Frozen embryo transfer	No association between vaccination status and timing and number of mature oocytes.	(35)
Myriam Safrai, et al. (2022)	Retrospective cohort study	Israel	42 vaccinated women and 42 controls	mRNA vaccine	N/A	Fresh embryo transfer	Vaccination does not affect IVF performance and outcomes from the early stage of oocyte development through to the early beginning of pregnancy.	(36)
Chana Adler Lazarovits, et al. (2022)	Prospective, observational study	Israel	99 female patients were vaccinated three times, 24 were vaccinated without the booster dose, 21 were convalescent, and 13 were unexposed	mRNA vaccine	N/A	Fresh embryo transfer	IVF outcomes are not affected by the mRNA vaccine, in particular the three-dose regimen.	(37)
Meng Dong, et al. (2022)	Prospective cohort study	China	735 infertile couples	Inactivated vaccines and recombinant subunit vaccines	<3 months: 33 cycles, 3-6 months: 103 cycles, >6 months: 38 cycles	Fresh and frozen embryo transfer	Vaccination status, types of vaccines, and intervals had no significant effects on the embryo quality and pregnancy rates in IVF.	(38)
Yixuan Wu, et al. (2022)	Retrospective cohort study	China	240 vaccinated women and 1343 controls	Inactivated vaccine	≤30 days: 27.5% women; 31-60 days: 38.4% women; ≥61days: 34.1% women.	Fresh embryo transfer	Vaccination has no detrimental effect on the number of oocytes retrieved, embryos suitable for transfer and blastocysts, rates of ongoing pregnancy implantation, biochemical pregnancy, clinical pregnancy and early miscarriage in women undergoing IVF treatment.	(39)
Jialyu Huang, et al. (2022)	Retrospective cohort study	China	146 vaccinated women and 584 controls	Inactivated vaccine	≤1 months: 37 women, 1-2 months: 42 women, >2 months: 71 women	Fresh embryo transfer	No significant associations were observed between vaccination and cycle characteristics or other laboratory and pregnancy outcomes.	(40)
Mingzhu Cao, et al. (2022)	Retrospective study	China	502 vaccinated women and 1589 controls	Inactivated vaccines	N/A	Frozen-thawed embryo transfer	Inactivated Covid-19 vaccines did not undermine live birth and neonatal outcomes of women planning for FET.	(41)
Wenhao Shi, et al. (2022)	Retrospective study	China	667 vaccinated women and 2385 controls	Inactivated vaccines	≤30 days: 35 women, 31-60 days: 58 women, 61-90 days: 105 women, and ≥91 days: 469 women.	Fresh embryo transfer	Receipt of the first inactivated COVID-19 vaccine dose 60 days or less before fertilization treatment is associated with a reduced rate of pregnancy.	(42)
Our study	Retrospective study	China	267 vaccinated women and 268 controls	Inactivated vaccines and recombinant subunit vaccines	7-182 days	Fresh embryo transfer	More MI oocytes and lower fertilization rate were observed in vaccinated women, especially in women with recombinant vaccine.	

N/A, not applicable.

## Discussion

The safety of vaccines against COVID-19 in women undergoing IVF/ICSI treatment is not well established. There were no placebo-controlled studies in women being vaccinated against COVID-19 in women undergoing IVF/ICSI treatment with regard to relevant safety endpoints for human reproduction. Only observational studies were reported. In addition, a comparison of the different vaccine types (mRNA vaccine, Adeno-viral based vaccine, inactivated vaccine types) is missing. We thus systematically analyzed safety data from our center and data coming from so far published observational studies. Oocyte maturation, fertilization rate, blastulation rate, implantation rate, clinical pregnancy rate, and miscarriage rate were the criteria to evaluate reproductive safety. Two types of vaccines against COVID-19 were mainly analyzed. For the mRNA vaccine no adverse signals were reported concerning fertilization rate, blastulation rate, implantation rate, clinical pregnancy rate, and miscarriage rate. Data on life birth rate – probably the most important hard clinical endpoint – and oocyte maturation, however, after mRNA vaccination were not reported so far. Thus, as of today, mRNA vaccines seem to be safe for women undergoing IVF/ICSI treatment, see **Table 6**.

In women being vaccinated with the Chinese inactivated vaccine, implantation rate, clinical pregnancy rate, and miscarriage rate were not affected. However, we saw a decreased fertilization rate and signs for impaired oocyte maturation in vaccinated women in women with recombinant and inactivated vaccine. Until now, the effect of COVID-19 vaccination on oocyte maturation was reported in nine studies testing mRNA vaccination and there was no difference in oocyte maturation between vaccinated and unvaccinated patients in all these studies (24, 30, 33–37, 40, 42). Safety data on adenovirus vector vaccines and recombinant subunit vaccines of COVID-19 in women undergoing ART treatment were limited. Only our study observed a sign of impaired oocyte maturation in women with recombinant vaccine especially, hence, further confirmation on this safety endpoint is required. The folliculogenesis process is complex and dynamic, which involves multiple endocrine cells and numerous signals that have been estimated to span > three months (43). Follicular cytokines in folliculogenesis are keys to reproductive success. They induce an immune-permissive, embryotropic environment that supports gametogenesis, fertilization, implantation, embryo development, and fetal growth (44). Anti-Müllerian hormone (AMH), produced by the granulosa cells of the ovarian follicles, is an indicator of a patient’s ability to respond quickly and efficiently to ovarian reserve (45). Being a member of transforming growth factor b (TGF-b) superfamily, AMH is involved in cytokine and growth factor network in the human body and responsible for the intercellular communications and immunological responses including inflammation. AMH may protect the ovary from inflammation by decreasing its own production in response to a rise in cytokines (46). The COVID-19 infection could activate a pro-inflammatory response and lead to cytokine storm (such as interferons, interleukins, chemokines, colony-stimulating factors, and TNF-alpha) and systemic inflammation (47). Vaccination might cause some of these inflammatory responses, which might also interfere with folliculogenesis, resulting in abnormal oocytes and fertilization (24). Clear evidence shows that after vaccination (for example, influenza vaccine), the “innate immune system”/”innate immunity” is activated and the vaccine is processed by antigen-presenting cells, which include macrophages and dendritic cells. Macrophages can produce some inflammatory molecules (cytokines), which is one of the important reasons why there is a local reaction after the vaccine at the site of injection. Mature dendritic cells migrate into regional lymph nodes, where they induce activation and clonal expansion of naïve CD4+ T-helper and CD8+ cytotoxic T cells. The activation and differentiation of naïve B cells are induced by antigen and CD4+ T-helper cells. Naïve B cells differentiate into memory B cells and antibody-secreting B cells. Long-term immunity is assured by memory B and T cells in the blood and lymph nodes as well as by long-living plasma cells and memory T cells in the bone marrow (48). These early inflammatory processes after vaccination might have an effect on folliculogenesis in the stimulated ovaries. Basic science studies are needed to confirm this hypothesis coming from our clinical data.

Vaccinating women in the third trimester of pregnancy offers the opportunity to provide early protection to infants through the transplacental transfer of maternal antibodies (49). Many studies have demonstrated the efficient transfer of pertussis antibodies across the placenta (50, 51). The WHO has recommended influenza vaccination during influenza season for all pregnant women since 2005 (3). Min Seo Kim et al. compared the safety of mRNA COVID-19 vaccines to influenza vaccines and demonstrated that the overall safety profile pattern suggests a lower risk of serious adverse events following immunization by mRNA vaccines compared to influenza vaccines (52).

However, limitations also exist in our single center observational study as well as in all other vaccination studies. We cannot fully exclude potential bias even though we made PSM before the study. Moreover, all studies only did first-term trimester follow-ups, whether long-term pregnancy complications and neonatal outcomes are affected needs to be investigated. Since all studies in the field are just observational studies, none of the studies can control for yet unknown confounding factors.

## Conclusion

Vaccination against COVID-19 in women undergoing ART treatment seems to be safe especially for women getting mRNA vaccines. The effects on oocyte maturation and fertilization rate of inactivated and recombinant COVID-19 vaccinations need further investigation and independent confirmation.

## Data availability statement

The original contributions presented in the study are included in the article/**Supplementary Material**. Further inquiries can be directed to the corresponding authors.

## Ethics statement

The studies involving human participants were reviewed and approved by Reproductive and Genetic Hospital of CITIC-Xiangya (approval number: LL-SC-2022). The patients/participants provided their written informed consent to participate in this study.

## Author contributions

BH and HC designed the study. HC collected the data. HC and XZ analyzed the data. HC, XZ, and BH wrote the manuscript. HC, GL, FG, and BH revised the manuscript. All authors contributed to the article and approved the submitted version
