# Supplementary material for: Safety of COVID-19 vaccination in women undergoing IVF/ICSI treatment - Clinical study and systematic review
Source: Front Immunol. 2023 Jan 11;13:1054273. doi: 10.3389/fimmu.2022.1054273 (PMC9876364; doi:10.3389/fimmu.2022.1054273)
Supplement: Supplementary file 1 [file DataSheet_1.docx]

Supplementary materials

Supplementary Figures

Supplementary figure S1. Flow chart.


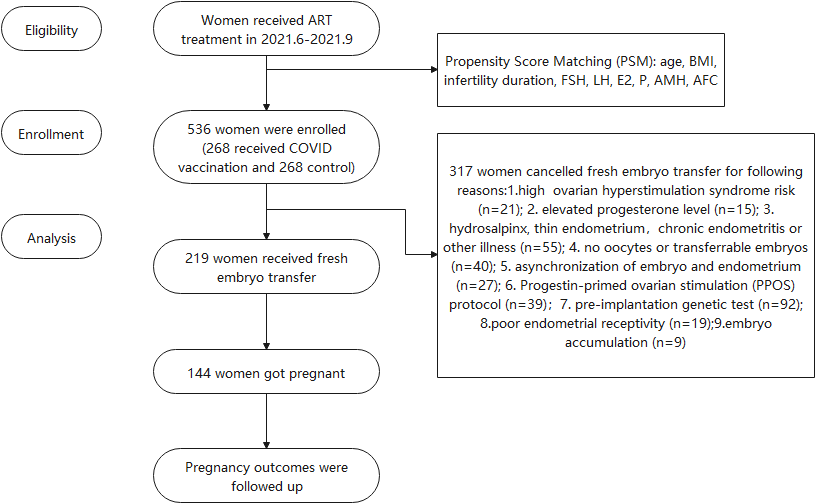


Supplementary Tables

Supplementary Table S1. Detail information of vaccine used in the current participants.

| Name | Type | Number |
| --- | --- | --- |
| 国药集团Sinopharm | Inactivated vaccine | 114 |
| 安徽智飞Anhui Zhifei Longcom Biopharmceutical Co., Ltd | recombinant subunit vaccine | 44 |
| 科兴Sinovac Research & Development Co., Ltd (Sinovac) | Inactivated vaccine | 104 |
| 深圳康泰（KCONVAC） | Inactivated vaccine | 5 |
| 康希诺 CanSinoBIO (CanSino) | Adenovirus vector vaccine | 1 |

**Principle of vaccines ^1^**

Inactivated COVID-19 Vaccines

All three vaccines use Vero cells to culture and grow COVID-19 virus and β-propionolactone to inactivate the virus while retaining antigenic components that induce immune responses. All three are adjuvanted with aluminum hydroxide to improve immunogenicity.

Recombinant Subunit COVID-19 Vaccine

The recombinant subunit COVID-19 vaccine (CHO cell) that has been approved for emergency use is manufactured by Anhui Zhifei Longcom Biopharmceutical Co., Ltd. This vaccine is made by recombining the S-protein receptor binding domain (RBD) gene with a gene in Chinese hamster ovary (CHO) cells. The CHO cells make RBD protein dimer that are purified and concentrated for use in producing the vaccine. Aluminum hydroxide adjuvant is added to improve immunogenicity.

Adenovirus Vectored COVID-19 Vaccine

The conditionally-approved adenovirus-5 vectored COVID-19 vaccine is manufactured by CanSinoBIO (CanSino). This vaccine is made by recombining the spike-glycoprotein (S protein) gene of COVID-19 virus into the genetic material of a replication-deficient human adenovirus-5. After injection of the vaccine, the recombinant adenovirus brings the S-protein gene into the person’s cells that then use the gene to make S-protein antigen, which induces an immune response in the person vaccinated.

Reference

1. Covid-Vaccine Technical Working Group. Technical Vaccination Recommendations for COVID-19 Vaccines in China (First Edition). China CDC Wkly. 2021;3(21):459-461. doi:10.46234/ccdcw2021.083

Supplementary Table S2. Laboratory outcomes in women after different time interval from inactivated vaccination to ART treatment

|  | ＜6 weeks (n=118) | ≥6 weeks  (n=105) | P value |
| --- | --- | --- | --- |
| No. of oocytes retrieved | 10·39±6·80 | 9·89±6·73 | 0·57 |
| No. of degenerated oocytes | 0·07±0·25 | 0·07±0·29 | 0·97 |
| No. of GV phase oocytes | 0·68±1·27 | 0·55±1·04 | 0·42 |
| No. of MI phase oocytes | 0·63±1·68 | 0·58±1·19 | 0·81 |
| No. of MII phase oocytes | 9·02±6·11 | 8·69±6·16 | 0·68 |
| Fertilization methods |  |  |  |
| IVF | 72·55 (74) | 69·23 (63) | 0·87 |
| ICSI | 19·61 (20) | 21·98 (20) |  |
| IVF+ICSI | 7·84 (8) | 8·79 (8) |  |
| Normal fertilization rate (%) | 63·35 (776/1225) | 62·68 (650/1037) | 0·74 |
| No. of 1PN zygotes | 0·38±0·60 | 0·37±0·58 | 0·90 |
| No. of 2PN zygotes | 5·14±5·49 | 5·31±5·58 | 0·63 |
| No. of ≥3PN zygotes | 0·54±1·01 | 0·67±0·97 | 0·33 |
| D3 embryo quality* |  |  |  |
| Fair | 4·73±3·90 | 4·57±3·84 | 0·76 |
| Good | 3·30±3·34 | 3·31±3·36 | 0·96 |
| 8C-I | 0·95±1·57 | 0·94±1·31 | 0·97 |
| D3 good quality embryo rate (%) | 41·06 (388/945) | 42·01 (347/826) | 0·68 |
| Blastocyst formation rate (%) |  |  |  |
| D5 | 38·75 (267/689) | 36·84 (210/570) | 0·48 |
| D6 | 37·12 (209/563) | 42·30 (195/461) | 0·09 |
| D7 | 28·85 (30/104) | 28·21 (33/117) | 0·91 |
| Good quality blastocyst rate (%)** |  |  |  |
| D5 | 16·85 (45/267) | 15·24 (32/210) | 0·63 |
| D6 | 31·58 (66/209) | 30·26 (59/195) | 0·77 |
| D7 | 33·33 (10/30) | 21·21 (7/33) | 0·27 |

GV: germinal vesicle, GV phase oocytes did not resume meiosis (immature oocytes); MI: metaphase I, oocytes in the middle of the first meiosis (immature oocytes); MII: metaphase II, oocytes in the middle of the second meiosis (mature oocytes); IVF: in vitro fertilization; ICSI: intracytoplasmic sperm injection; PN: pronucleus (only 2PN means normal fertilization); D5: day 5 after fertilization; D6: day 6 after fertilization; D7: day 7 after fertilization.

* D3 embryo quality: day 3 after fertilization. Fair quality embryo: embryo grade ＜7C-II，good quality embryo:：embryo grade ≥7C-II.

**Good quality blastocyst: ≥4BB

Supplementary Table S3. Laboratory outcomes in women after different time interval from recombinant vaccination to ART treatment

|  | ＜6 weeks (n=21) | ≥6 weeks (n=23) | P value |
| --- | --- | --- | --- |
| No. of oocytes retrieved | 12·76±9·78 | 12·22±6·95 | 0·83 |
| No· of degenerated oocytes | 0·14±0·36 | 0·04±0·21 | 0·26 |
| No· of GV phase oocytes | 0·86±2·61 | 0·74±1·91 | 0·86 |
| No· of MI phase oocytes | 0·52±0·87 | 1·00±1·41 | 0·19 |
| No· of MII phase oocytes | 11·24±9·02 | 10·43±6·40 | 0·73 |
| Fertilization methods |  |  |  |
| IVF | 88·24 (15) | 76·19 (16) | 0·52 |
| ICSI | 11·76 (2) | 19·05 (4) |  |
| IVF+ICSI | 0 (0) | 4·76 (1) |  |
| Normal fertilization rate (%) | 62·31 (167/268) | 56·58 (159/281) | 0·17 |
| No· of 1PN zygotes | 0·24±0·44 | 0·74±1·01 | 0·04 |
| No· of 2PN zygotes | 9·90±8·30 | 8·65±6·44 | 0·57 |
| No· of ≥3PN zygotes | 1·43±1·63 | 0·91±1·28 | 0·24 |
| D3 embryo quality* |  |  |  |
| Fair | 5·14±4·86 | 4·57±3·70 | 0·65 |
| Good | 4·62±4·81 | 2·91±2·94 | 0·15 |
| 8C-I | 2·05±2·44 | 1·00±1·51 | 0·09 |
| D3 good quality embryo rate (%) | 47·32 (97/205) | 38·95 (67/172) | 0·10 |
| Blastocyst formation rate (%) |  |  |  |
| D5 | 45·26 (62/137) | 30·14 (44/146) | ＜0·01 |
| D6 | 35·92 (37/103) | 31·90 (37/116) | 0·53 |
| D7 | 22·22 (2/9) | 33·33 (3/9) | 1·00 |
| Good quality blastocyst rate (%)** |  |  |  |
| D5 | 25·81 (16/62) | 9·09 (4/44) | 0·03 |
| D6 | 35·14 (13/37) | 8·11 (3/37) | 0·01 |
| D7 | 0 (0/2) | 100 (3/3) | 0·10 |

GV: germinal vesicle, GV phase oocytes did not resume meiosis (immature oocytes); MI: metaphase I, oocytes in the middle of the first meiosis (immature oocytes); MII: metaphase II, oocytes in the middle of the second meiosis (mature oocytes); IVF: in vitro fertilization; ICSI: intracytoplasmic sperm injection; PN: pronucleus (only 2PN means normal fertilization); D5: day 5 after fertilization; D6: day 6 after fertilization; D7: day 7 after fertilization·

* D3 embryo quality: day 3 after fertilization· Fair quality embryo: embryo grade ＜7C-II，good quality embryo:：embryo grade ≥7C-II·

**Good quality blastocyst: ≥4BB

Supplementary Table S4. Pregnancy outcomes in women after different time interval from inactivated vaccination to ART treatment

|  | ＜6 weeks (n=37) | ≥6 weeks (n=40) | P value |
| --- | --- | --- | --- |
| EM thickness before ET (mm) | 12·07±2·11 | 12·41±1·67 | 0·44 |
| Mean number of embryos transferred | 1·73±0·45 | 1·65±0·48 | 0·46 |
| Day3 embryo | 1·88±0·33 | 1·88±0·33 | 0·96 |
| Blastocyst | 1·31±0·48 | 1·14±0·36 | 0·32 |
| Good-quality embryo transferred rate (%) | 78·13(50/64) | 81·82 (54/66) | 0·60 |
| Clinical pregnancy rate (%) | 54·05 (20/37) | 65·00 (26/40) | 0·33 |
| Implantation rate (%) | 39·06 (25/64) | 51·52 (34/66) | 0·15 |
| Miscarriage rate (%) | 2·70 (1/37) | 2·50 (1/40) | 0·96 |

Em: endometrium; ET: embryo transfer

Supplementary Table S5. Pregnancy outcomes in women after different time interval from recombinant vaccination to ART treatment

|  | ＜6 weeks (n=4) | ≥6 weeks (n=11) | P value |
| --- | --- | --- | --- |
| EM thickness before ET (mm) | 10·55±1·52 | 13·15±1·78 | 0·23 |
| Mean number of embryos transferred | 2·00±0 | 1·82±0·41 | 0·40 |
| Day3 embryo | 2·0±0 | 1·88±0·35 | 0·51 |
| Blastocyst | 0 | 1·25±0·50 | ____ |
| Good-quality embryo transferred rate (%) | 75·00 (6/8) | 75·00 (15/20) | 1·00 |
| Clinical pregnancy rate (%) | 25·00 (1/4) | 90·90 (10/11) | 0·03 |
| Implantation rate (%) | 12·50 (1/8) | 65·00 (13/20) | 0·03 |
| Miscarriage rate (%) | 0 (0/4) | 18·18 (2/11) | 1·00 |

Em: endometrium; ET: embryo transfer
